# Efficacy and Acceptability of Different Auxiliary Drugs in Pediatric Sevoflurane Anesthesia: A Network Meta-analysis of Mixed Treatment Comparisons

**DOI:** 10.1038/srep36553

**Published:** 2016-11-10

**Authors:** Wuchao Wang, Panchuan Huang, Weiwei Gao, Fangli Cao, Mingling Yi, Liyong Chen, Xiaoli Guo

**Affiliations:** 1 Department of Anesthesiology, Pain Diagnosis and Treatment Center, Research Institute of Surgery & Daping Hospital, Third Military Medical University, Daping, Chongqing, 400042, China

## Abstract

Emergence agitation preventive medicine should be combined with pediatric anesthesia because of the high frequency of emergence agitation. However, it is challenging to determine the most appropriate medication that can be introduced into pediatric anesthesia for the sake of emergence agitation prevention. We reviewed and retrieved the data from PubMed and Embase. Various medications were assessed based on several endpoints including Emergence agitation outcomes (EA), postoperative nausea and vomiting (PONV), the number of patients who required analgesic (RA), pediatric anesthesia emergence delirium (PAED), the extubation time, the emergency time and the duration of post-anesthesia care unit (PACU) stay. Both traditional and network meta-analysis were carried in this study. A total of 45 articles were complied with the selection criteria and the corresponding articles were reviewed. Fentanyl demonstrated the highest cumulative ranking probability which was followed by those of ketamine and dexmedetomidine with respect to EA and PAED. When PONV and RA were concerned together, clonidine exhibited the highest cumulative ranking probability compared to other medications. Our study suggested that dexmedetomidine perhaps is the most appropriate prophylactic treatment which can be introduced into anesthesia for preventing emergence agitation.

Sevoflurane has been introduced into clinical practices as an inhaled volatile anesthetic since 1992. This medication is particularly effective for inhalation induction and maintaining the effects of general anesthesia on pediatric patients due to its inherent stability, minimal respiratory pungency and minimal blood-gas partition coefficient^1^. Another advantage of sevoflurane is its ability to rapidly induce anesthetic effects in a controllable manner once injected.

Unfortunately, postoperative behavioral disturbance was predominantly observed in patients who received pediatric surgeries accompanied by sevoflurane as anesthetic. Another major issue caused by sevoflurane is the significant increase in the incidence of emergence agitation (EA). For instance, the incidence of emergence agitation was increased from 12–13% to 56% when sevoflurane was introduced as the main agent^2^,^3^.

Emergence agitation resulted from general anesthesia is usually characterized by either disorientation or abnormal excitation during the early stage of patient recovery. However, more severe symptoms such as sympathetic activation and arrhythmia are likely to be observed, which may further impede the recovery of patients. Some researches argued that the toxicity of sevoflurane may affect the central nervous system and trigger EA, while others suggested that other factors including age may contribute to EA^4^. Since sevoflurane is likely to induce EA in certain circumstances, prophylactic medicine has been introduced into sevoflurane in order to enhance the recovery of patients and reduce the risk of postoperative behavioral disturbance. Conventional prophylactic medicine includes sedative-hypnotic, opioid receptor agonist and narcotic analgesic and they have been introduced into sevoflurance in clinical practices. On the other hand, treatments for preventing EA include midazolam, dexmedetomidine, clonidine, ketamine, propofol and fentanyl and they appear to have significant difference in pharmacological characteristics. As a result, the effectiveness and safety of these treatments should be verified in clinical practices.

This study enabled us to compare the effectiveness and safety of placebo, midazolam, dexmedetomidine, clonidine, ketamine, propofol and fentanyl which are commonly introduced as prophylactic treatments. We incorporated various endpoints in our study so that both direct and indirect comparison can be comprehensively achieved.

## Materials and Methods

Two phases were involved in this study. Phase one was collecting all the articles about the efficacy and safety of seven auxiliary medications that are introduced into pediatric sevoflurane anesthesia. Phase two was meta-analysis on a select group of these techniques.

### Search strategy

Articles complied with the selection criteria were thoroughly searched, including PubMed, Embase and other databases. The following keywords and searching terms including their corresponding synonyms were used to retrieve the corresponding articles according to standard PICOS (population, intervention, comparison, outcome, study design) criteria: pediatric anesthesia (population), clonidine, dexmedetomidine, fentanyl, ketamine, midazolam, propofol (intervention and comparison) and randomized controlled trial (study design), emergence agitation (primary outcome).

### Inclusion and exclusion criteria

Literature inclusion criteria: (1) researching type: randomized controlled trials; (2) researching objects: children between the age of six months and fourteen years who received sevoflurane as anesthetic (3) interventions: single or mixed clonidine, dexmedetomidine, fentanyl, ketamine, midazolam, propofol; (4) outcomes contain at least one of the followings: EA, postoperative nausea and vomiting (PONV), the number of patients who required analgesic, pediatric anesthesia emergence delirium (PAED), the extubation time, the emergency time and the duration of PACU stay. Literature exclusion criteria: (1) non-randomized controlled trials; (2) research objects were not complied with the inclusion criteria; (3) literatures which were not written in English; (4) duplicated literatures which were published by the same author; (5) literatures in which data integrity cannot be guaranteed. A Jadad Scale table concerning randomization, blinding and withdraw was used as an appendix to qualify the included papers (Table S1).

### Outcome measures and data extraction

Data extraction was performed using a standard approach: two researchers (W. C. Wang and P. Huang) extract the corresponding data from literatures independently including the sample size and data integration was also carried out for each study. The number of paper included varied between researchers, and difference in data extraction was used for correction. Any disagreement or different opinions with respect to data extraction and integration was resolved by a third researcher (X. L. Guo).

### Statistical analysis

First, we accomplished a conventional meta-analysis on the selected data. Odds ratios (OR) were selected as the appropriate statistics for comparing binary outcomes whereas standardized mean difference (SMD) were selected for comparing continuous outcomes. Apart from that, the 95% CI were also obtained in order to assess the precision of the corresponding statistics. Heterogeneity across studies was assessed by the statistic of *I*^*2*^ and significant heterogeneity was presented if *I*^*2*^ > 50%. The fixed-effect model was implemented if studies are homogeneous in nature (*P*-value of heterogeneity >0.05). By contrast, the random-effects were chosen in the case of significant heterogeneity (*P*-value for heterogeneity <0.05).

Moreover, the network meta-analysis was conducted in the same manner and the surface under the cumulative ranking curve (SUCRA) in order to rank the corresponding interventions. SUCRA, a transformation of the mean rank, provides a hierarchy of treatments and accounts for the location and variance of clinical outcomes. Higher accumulative SUCRA values indicate better treatment ranks, which is equal to 1 when the treatment is certain to be the best.

## Results

### Literature search results

**Figure 1:**
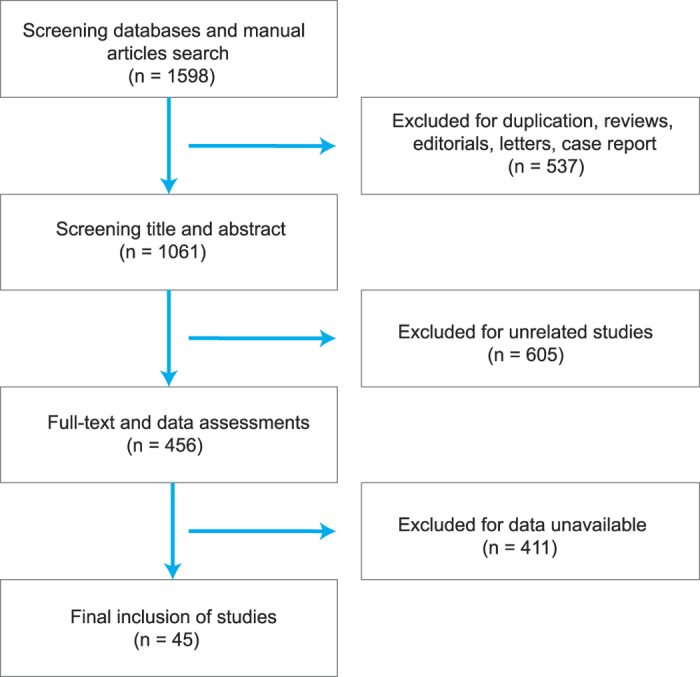
The flow chart of literature selection.

We identified a total of 1,598 publications and 537 of them were removed since they are either duplicated literatures, comments, letters and case reports. Another 605 publications were removed since they were not related to the research topic and 411 of the remaining articles contain incomplete data. As a result of this, 45 articles published from 1999 to 2015 were complied with the selection criteria (Fig. 1)^5^,^6^,^7^,^8^,^9^,^10^,^11^,^12^,^13^,^14^,^15^,^16^,^17^,^18^,^19^,^20^,^21^,^22^,^23^,^24^,^25^,^26^,^27^,^28^,^29^,^30^,^31^,^32^,^33^,^34^,^35^,^36^,^37^,^38^,^39^,^40^,^41^,^42^,^43^,^44^,^45^,^46^,^47^,^48^,^49^. A total of 4,032 cases were included and the detailed baseline characteristics of the included studies were displayed in Table 1. A Jadad Scale table concerning randomization, blinding and withdraw was used as an appendix to qualify the included papers (Table S1).

### Conventional meta-analysis

**Figure 2:**
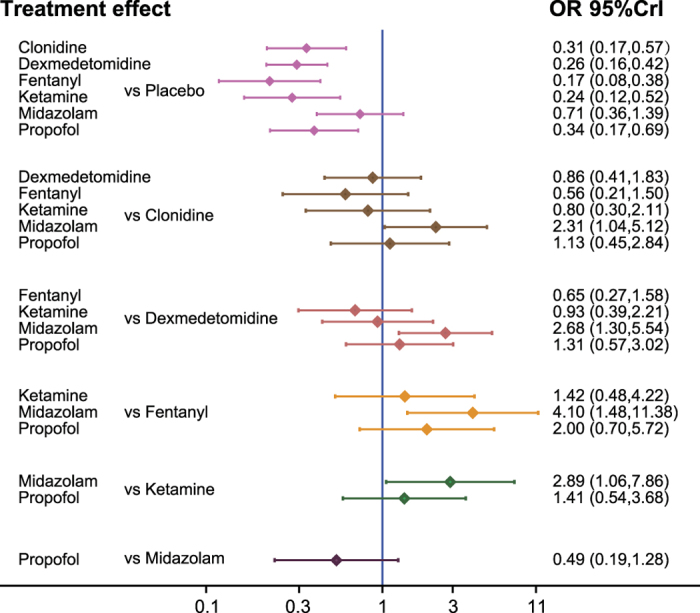
The forest plot of different treatment on emergence agitation from network meta-analysis.

We carried out conventional meta-analysis to compare the efficacy and safety of seven auxiliary medications that are introduced into pediatric sevoflurane anesthesia (Table 2). Clonidine, dexmedetomidine, fentanyl and ketamine and propofol significantly reduced the risk of EA (Fig. 2). The same approach was adopted to evaluate the relative safety of these auxiliary medications compared to placebo. Both clonidine and dexmedetomidine were associated with a decrease in the risk of PONV. Furthermore, patients with dexmedetomidine experienced a reduced risk of sedative. Fentanyl exhibited less favorable results than the placebo with respect to PONO, the emergency time and the duration of PACU stay. However, Fentanyl showed compelling results with respect to RA and PAED. Ketamine exhibited convincing results in both PAED and the emergency time. We also observed that patients treated with clonidine, dexmedetomidine, fentanyl and midazolam and propofol exhibited significantly longer emergency response time compared to placebo. Patients treated with propofol were associated with a downward trend of RA and PAED.

### Network meta-analysis

We also carried out pair wise comparisons among these medications through network meta-analysis Table 3: patients treated with clonidine, dexmedetomidine, fentanyl, ketamine and propofol were less likely to have EA. Fentanyl exhibited the least favorable results with respect to PONV compared to the other six auxiliary medications whereas clonidine and dexmedetomidine exhibited more compelling results than placebo. Additionally, dexmedetomidine, fentanyl, ketamine and midazolam were less likely to result in sedatives use compared to placebo. Our study also demonstrated that dexmedetomidine, fentanyl and ketamine significantly reduced the average PAED in comparison to placebo and dexmedetomidine appeared to be more effective than clonidine with respect to PAED.

Besides, we compared the average extubation and emergency time for determine the overall safety of these medications. Patients treated with dexmedetomidine exhibited significantly longer extubation time compared to those who were given placebo. On the other hand, patients treated with clonidine, dexmedetomidine, fentanyl and ketamine and midazolam exhibited significantly shorter emergency time compared to those treated with propofol, and ketamine group had significantly shorter average emergency time compared to the midazolam group. The comparison of duration for the corresponding treatments revealed that both clonidine and fentanyl demonstrated relatively longer duration of PACU stay compared to placebo whereas such a figure in the propofol group is significantly shorter than that in the clonidine group.

**Figure 3:**
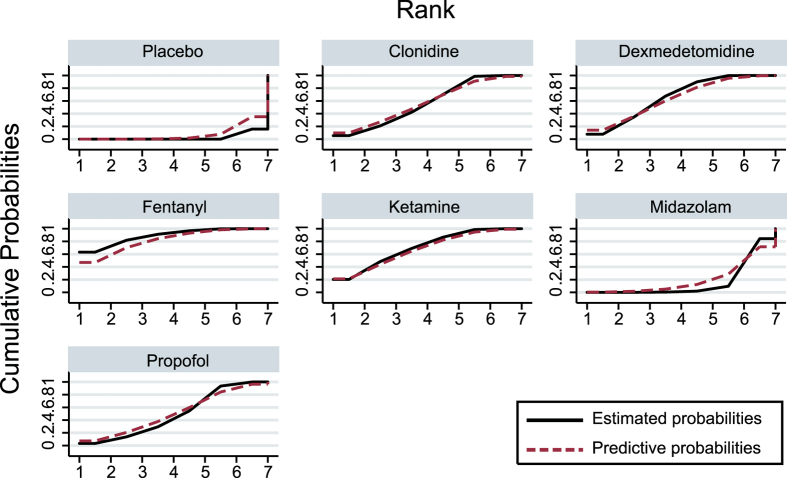
The cumulative ranking probabilities of different treatment on emergence agitation.

The corresponding SUCRA values of seven pediatric sevoflurane anesthesia auxiliary medications with respect to each efficacy and safety endpoint were illustrated in Table 4, Fig. 3 and Figs S1a–S6a. Fentanyl had the highest cumulative ranking probability with respect to EA and PAED (EA, 88.8%; PAED, 83.9%) whereas both ketamine and dexmedetomidine demonstrated robust results with respect to EA (70.5% and 66.7%, respectively); clonidine exhibited the most compelling SUCRA values with respect to PONV and RA (PONV, 91.6%, RA, 75.0%) and ketamine ranked the best with respect to the emergency time (96.0%). More importantly, placebo exhibited the highest cumulative ranking probability with respect to the extubation time and PACU, therefore other medications may trigger several adverse effects which are reflected by longer extubation time and PACU (Extubation Time, 80.7%; the PACU, 92.2%).

## Discussion

In current study, we conducted a network meta-analysis to compare the relative efficacy and safety of six prophylactic treatments including clonidine, dexmedetomidine, fentanyl, ketamine, midazolam and propofol. Our results showed that fentanyl, ketamine, and dexmedetomidine are significantly associated with a lower risk of EA and PAED together with enhanced effectiveness compared to the placebo. It appears that dexmedetomidine is more appropriate than others and such a conclusion is supported by Fang *et al*. reporting that dexmedetomidine was the most appropriate medication with respect to EA prevention^50^.

One potential explanation for above conclusion is that dexmedetomidine is an α(2)-adrenoceptor agonist with several analgesic, anxiolytic and sedative properties. It is suspected that these properties may enhance the hemodynamic stability, hence contributing to risk reduction of EA^51^,^52^. It is acknowledged that pain relief medicine is able to reduce anesthesia-related EA effectively^23^,^29^,^53^. However, some researchers argued that the use of general analgesic is not effective in reducing the risk of EA^54^. Dahmani *et al*. demonstrated that the sedation triggered by dexmedetomidine played a key role in reducing the risk of EA during the recovery period^55^. Therefore, we suspect that the reduction in the risk of EA is likely to be triggered by the analgesic and anxiolytic roles of dexmedetomidine. Apart from that, dexmedetomidine has somehow neuroprotective effects which are able to reduce neurocognitive impairment resulted from anesthetics^56^. Meanwhile, Robert *et al*. reported that the neuroprotective effect of dexmedetomidine resulted from the increase of expression levels of Mdm2 and Bcl-2, up-regulating the neurotrophic factor-Cyclic AMP response element-binding protein (BDNF-CREB) and activating the ERK signaling pathways^57^,^58^,^59^.

This study demonstrates that fentanyl is particularly more effective than dexmedetomidine in reducing the risk of EA and PAED. As suggested by Fenmei *et al*., fentanyl is able to reduce the risk of EA in a non-specific way regardless of its undiscovered relationship with postoperative pain and EA^60^,^61^,^62^. This may be explained by the fact that fentanyl has a durable analgesic and sedative effect. However, fentanyl has excitatory effects on the gastrointestinal smooth muscle and both patients in the fentanyl group are more likely to experience PONV and RA compared to those in the dexmedetomidine group. Furthermore, the effect of ketamine on risk reduction of PAED and EA is almost equal to that contributed by dexmedetomidine which is consistent with a study conducted by Dahmani *et al*.^55^ ketamine is an aspartate receptor antagonist which not only exhibits similar sedative and hypnotic effects to those of dexmedetomidine but also contain strong analgesic effects^63^,^64^,^65^,^66^.

This study is a network meta-analysis which compares different types of prophylactic treatments including clonidine, dexmedetomidine, fentanyl, ketamine, midazolam, and propofol. However, some limitations should be further addressed by future researchers due to the nature of network meta-analysis. For instance, there may be significant variations with respect to design, sample size and patient selection which cannot be incorporated by our network meta-analysis. Apart from that, the unequal number of interventions for each endpoint did not enable us to carry out a cluster analysis. In summary, our findings suggested that dexmedetomidine should be considered as the most appropriate prophylactic treatment that can be introduced into sevoflurane anesthesia. We recommend researchers to carry out specific following-up studies so that the long-term effects of these interventions can be discovered.

## Additional Information

**How to cite this article**: Wang, W. *et al*. Efficacy and Acceptability of Different Auxiliary Drugs in Pediatric Sevoflurane Anesthesia: A Network Meta-analysis of Mixed Treatment Comparisons. *Sci. Rep*. **6**, 36553; doi: 10.1038/srep36553 (2016).

**Publisher’s note:** Springer Nature remains neutral with regard to jurisdictional claims in published maps and institutional affiliations.

## Supplementary Material

Supplementary Information

## Figures and Tables

**Table 1:** Main characteristics of included studies.

ID, author, year	Age	Premedication	Analgesia	Intervention	Size	Endpoints						
						1	2	3	4	5	6	7
01 Lundblad 2015	18 mo–8yr	None	Ilioinguinal/Iliohypogastric Nerve Blocks	Dexmedetomidine	22	√						
				Placebo	21	√						
02 Costi 2015	1–12yr	Midazolam: 0.5 mg/kg or None	None	Propofol	109	√					√	√
				Placebo	109	√					√	√
03 Sheta 2014	3–6yr	Dexmedetomidine	Paracetamol: 30–40 mg/kg	Dexmedetomidine	36	√		√			√	
		Midazolam		Midazolam	36	√		√			√	
04 Kim 2014	1–5yr	None	Caudal Block	Dexmedetomidine	20	√		√				
				Placebo	20	√		√				
05 Bortone 2014	2–11yr	Midazolam: 0.5 mg/kg	Penile Block or Ilio-inguinal/ Iliohypogastric Block or Caudal Block	Clonidine	29	√					√	√
				Fentanyl	29	√					√	√
				Placebo	29	√					√	√
06 Kim 2013	18–72mo	None	Caudal Block	Propofol	69		√	√	√		√	√
				Fentanyl	66		√	√	√		√	√
				Placebo	70		√	√	√		√	√
07 Chen 2013	2–7yr	None	None	Dexmedetomidine	28	√	√					√
				Ketamine	28	√	√					√
				Placebo	28	√	√					√
08 Meng 2012	5–14yr	Midazolam: 40 μg/kg	None	Dexmedetomidine	40	√	√	√		√	√	√
				Dexmedetomidine	40	√	√	√		√	√	√
				Placebo	40	√	√	√		√	√	√
09 Lili 2012	3–7yr	None	None	Dexmedetomidine	30	√						
				Placebo	30	√						
10 Akin 2012	2–9yr	Midazolam	None	Midazolam	45	√		√		√		
		Dexmedetomidine		Dexmedetomidine	45	√		√		√		
11 Pestieau 2011	6 mo–6yr	None	None	Dexmedetomidine	28	√		√			√	
				Dexmedetomidine	23	√		√			√	
				Fentanyl	23	√		√			√	
				Placebo	27	√		√			√	
12 Ozcengiz 2011	3–9yr	Dexmedetomidine	None	Dexmedetomidine	25	√						
		Midazolam		Midazolam	25	√						
		Placebo		Placebo	25	√						
13 Ghosh 2011	1–5yr	None	Caudal Epidural Block	Clonidine	30	√						
				Clonidine	30	√						
				Placebo	30	√						
14 Sato 2010	1–9yr	None	Acetaminophen: 40 mg/kg or Diclofenac: 1 mg/kg	Dexmedetomidine	39	√	√					
				Placebo	42	√	√					
15 Rampersad 2010	1–5yr	Midazolam: 0.5 mg/kg	Acetaminophen: 40 mg/kg	Fentanyl	75		√					
				Placebo	79		√					
16 Patel 2010	2–10yr	None	Acetaminophen: 30–40 mg/kg	Dexmedetomidine	61			√	√	√	√	
				Fentanyl	61			√	√	√	√	
17 Lee 2010	2–14yr	Atropine: 0.01 mg/kg	None	Ketamine	30	√	√			√		
				Ketamine	30	√	√			√		
				Placebo	30	√	√			√		
18 Lee 2010	3–8yr	Thiopental Sodium: 1 mg/kg	Ketorolac: 1 mg/kg	Propofol	44	√	√			√		√
				Placebo	44	√	√			√		√
19 Inomata 2010	2–6yr	None	Field Block	Fentanyl	45				√	√		
				Fentanyl	48				√	√		
				Placebo	46				√	√		
20 Al-Zaben 2010	1–12yr	None	None	Dexmedetomidine	24			√		√		
				Placebo	24			√		√		
21 Saadawy 2009	1–6yr	None	Caudal Block	Dexmedetomidine	30	√	√					
				Placebo	30	√	√					
22 Tsai 2008	1–10yr	Midazolam: 0.2 mg/kg	None	Propofol	20	√						√
				Ketamine	20	√						√
				Placebo	20	√						
23 Abu-Shahwan 2008	2–7yr	None	None	Propofol	42	√			√		√	√
				Placebo	42	√			√		√	√
24 Tazeroualti 2007	1–6yr	Midazolam	Penile Block	Midazolam	20	√	√				√	
		Clonidine		Clonidine	20	√	√				√	
		Clonidine		Clonidine	20	√	√				√	
25 Kain 2007	2–10yr	Midazolam	None	Midazolam	99	√						
		None		Placebo	98	√						
26 Breschan 2007	6 mo–5yr	Midazolam	Caudal Blocks or penile blocks or local infiltration	Midazolam	57	√						
				Midazolam	58	√						
27 Aouad 2007	2–6yr	Midazolam: 0.5 mg/kg	Paracetamol: 15 mg/kg and dexamethasone: 1 mg/kg	Propofol	41	√			√		√	√
				Placebo	36	√			√		√	√
28 Almenrader 2007	1–6yr	Midazolam	Peripheral Nerve Block or Caudal Block	Midazolam	34	√						
		Clonidine		Clonidine	30	√						
29 Abu-Shahwan 2007	4–7yr	Acetaminophen: 30 mg/kg and Midazolam: 0.5 mg/kg	Ketorolac 1 mg/kg	Ketamine	42	√			√		√	√
				Placebo	42	√			√		√	√
30 Lankinen 2006	1–7yr	None	Alfentanil: 20 μg/kg and Diclofenac: 1 mg/kg	Clonidine	24	√	√				√	√
				Placebo	26	√	√				√	√
31 Isik 2006	18 mo–10yr	None	None	Dexmedetomidine	21	√	√				√	√
				Placebo	21	√	√				√	√
32 Dalens 2006	6 mo–8yr	None	None	Ketamine	33	√	√					
				Placebo	28	√	√					
33 Tesoro 2005	1–5yr	Midazolam: 0.5 mg/kg	Regional or Central block	Clonidine	91	√	√				√	
				Placebo	78	√	√				√	
34 Shukry 2005	1–10yr	None	None	Dexmedetomidine	23	√				√		√
				Placebo	23	√				√		√
35 Guler 2005	3–7yr	Acetaminophen: 15 mg/kg	None	Dexmedetomidine	30	√	√			√	√	
				Placebo	30	√	√			√	√	
36 Ibacache 2004	1–10yr	None	Caudal Block	Dexmedetomidine	30	√					√	√
				Dexmedetomidine	30	√					√	√
				Placebo	30	√					√	√
37 Demirbilek 2004	2–7yr	Midazolam: 0.5 mg/kg	Acetaminophen or Paracetamol: 30 mg/kg	Fentanyl	30	√	√			√	√	
				Placebo	30	√	√			√	√	
38 Binstock 2004	2–10yr	Fentanyl	Caudal Block	Fentanyl	27		√				√	√
		Fentanyl		Fentanyl	24		√				√	√
				Placebo	26		√				√	√
39 Bergendahl 2004	1–11yr	Midazolam and Atropine: 40 μg/kg	Fentanyl: 2.5 μg/kg	Midazolam	52	√	√					
		Clonidine and Atropine: 40 μg/kg		Clonidine	48	√	√					
40 Cravero 2003	18 mo–10yr	None	None	Fentanyl	16	√						
				Placebo	16	√						
41 Bock 2002	3–8yr	Midazolam: 0.4 mg/kg	Caudal Epidural Block	Clonidine	18	√						
				Clonidine	18	√						
				Placebo	18	√						
42 Kulka 2001	2–7yr	Midazolam: 0.5 mg/kg	Penile Block	Clonidine	20	√	√	√		√		
				Placebo	20	√	√	√		√		
43 Finkel 2001	6 mo–5yr	None	Acetaminophen: 40 mg/kg	Fentanyl	51	√	√					√
				Fentanyl	50	√	√					√
				Placebo	49	√	√					√
44 Galinkin 2000	9 mo–6yr	Midazolam: 0.5 mg/kg and Acetaminophen: 10 mg/kg	None	Fentanyl	64	√						√
				Placebo	69	√						√
45 Viitanen 1999	1–3yr	Midazolam	Acetaminophen: 20 mg/kg	Midazolam	30	√	√	√			√	√
		Placebo		Placebo	30	√	√	√			√	√

*Age: mo-month; yr-year; Endpoints: 1-emergence agitation; 2-postoperative nausea and vomiting; 3-requiring an analgesic; 4-pediatric anesthesia emergence delirium; 5-extubation time; 6-emergency time; 7-duration of postanesthesia care unit stay.

**Table 2:** Pair-wise meta-analyses of direct comparisons between the six drugs and placebo.

Endpoints	Direct comparisons	N	*I*^*2*^	*P*_*H*_ values	OR (95% CI)	*P*_*OR*_ values
**EA**	Clonidine vs. Placebo	461	0.657	0.012	0.332 (0.146, 0.754)	**0.008**
	Dexmedetomidine vs. Placebo	826	0.183	0.259	0.244 (0.160, 0.372)	**<0.001**
	Fentanyl vs. Placebo	483	0.000	0.528	0.233 (0.133, 0.406)	**<0.001**
	Ketamine vs. Placebo	331	0.563	0.058	0.248 (0.102, 0.605)	**0.002**
	Midazolam vs. Placebo	307	0.665	0.051	0.727 (0.235, 2.248)	0.579
	Propofol vs. Placebo	507	0.511	0.085	0.351 (0.178, 0.692)	**0.002**
**PONV**	Clonidine vs. Placebo	259	0.000	0.777	0.282 (0.094, 0.844)	**0.024**
	Dexmedetomidine vs. Placebo	419	0.000	0.595	0.438 (0.224, 0.857)	**0.016**
	Fentanyl vs. Placebo	577	0.139	0.326	3.154 (1.578, 6.303)	**0.001**
	Ketamine vs. Placebo	207	0.000	0.788	0.828 (0.394, 1.742)	0.619
	Midazolam vs. Placebo	60	0.000	0.000	0.097 (0.005, 1.877)	0.123
	Propofol vs. Placebo	227	0.126	0.285	1.010 (0.322, 3.168)	0.285
**RA**	Clonidine vs. Placebo	40	0.000	0.000	0.054 (0.003, 1.044)	0.053
	Dexmedetomidine vs. Placebo	286	0.324	0.218	0.128 (0.048, 0.339)	**<0.001**
	Fentanyl vs. Placebo	186	0.359	0.212	0.094 (0.016, 0.555)	**0.009**
	Midazolam vs. Placebo	60	0.000	0.000	0.187 (0.009, 4.062)	0.286
	Propofol vs. Placebo	139	0.000	0.000	0.046 (0.006, 0.356)	0.003
		**N**	***I***^***2***^	***P***_***H***_ **values**	**SMD (95% CI)**	***P***_***SMD***_ **values**
**PAED**	Fentanyl vs. Placebo	275	0.848	0.010	−1.251 (−1.936, −0.565)	**<0.001**
	Ketamine vs. Placebo	84	0.000	0.000	−5.435 (−8.051, −2.818)	**<0.001**
	Propofol vs. Placebo	300	0.509	0.131	−1.044 (−1.396, −0.691)	**<0.001**
**Extubation**	Clonidine vs. Placebo	40	0.000	0.000	0.424 (−0.203, 1.051)	0.185
**Time**	Dexmedetomidine vs. Placebo	274	0.910	0.000	0.837 (−0.064, 1.738)	0.069
	Fentanyl vs. Placebo	199	0.000	0.575	0.230 (−0.061, 0.521)	0.121
	Ketamine vs. Placebo	90	0.000	0.000	0.221 (−0.218, 0.661)	0.324
	Propofol vs. Placebo	88	0.000	0.000	0.379 (−0.042, 0.801)	0.078
**Emergency**	Clonidine vs. Placebo	277	0.077	0.338	0.394 (0.140, 0.648)	**0.002**
**Time**	Dexmedetomidine vs. Placebo	390	0.867	0.015	0.754 (0.149, 1.359)	**0.015**
	Fentanyl vs. Placebo	381	0.886	0.000	0.821 (0.163, 1.479)	**0.014**
	Ketamine vs. Placebo	84	0.000	0.000	−0.651 (−1.091, −0.212)	**0.004**
	Midazolam vs. Placebo	60	0.000	0.000	0.843 (0.314, 1.372)	**0.002**
	Propofol vs. Placebo	518	0.000	0.000	0.804 (0.624, 0.983)	**<0.001**
**Duration of PACU Stay**	Clonidine vs. Placebo	108	0.000	0.959	0.213 (−0.166, 0.591)	0.270
	Dexmedetomidine vs. Placebo	354	0.896	0.000	0.672 (−0.052, 1.395)	0.069
	Fentanyl vs. Placebo	554	0.470	0.109	0.314 (0.072, 0.555)	**0.011**
	Ketamine vs. Placebo	180	0.948	0.000	0.627 (−0.799, 2.053)	0.389
	Midazolam vs. Placebo	60	0.000	0.000	0.435 (−0.077, 0.947)	0.096
	Propofol vs. Placebo	646	0.741	0.002	0.119 (−0.202, 0.439)	0.468

*N: number of studies; H: heterogeneity; OR: odds ratio; CI: confidence interval; SMD: standard mean difference; EA: emergence agitation; PONV: postoperative nausea and vomiting; RA: requiring an analgesic; PAED: pediatric anesthesia emergence delirium.

**Table 3:** The efficacy (emergence agitation) and tolerability (PONV, RA, PAED, extubation time, emergency time, duration of PACU stay) of six treatments according to the network meta-analysis using odds ratios (ORs) or standard mean differences (SMDs) and corresponding 95% credible interval (CrI).

**Placebo**	**0.31 (0.17, 0.57)**	**0.26 (0.16, 0.42)**	**0.17 (0.08, 0.38)**	**0.24 (0.12, 0.52)**	**0.71 (0.36, 1.39)**	**0.34 (0.17, 0.69)**
	**Clonidine**	0.86 (0.41, 1.83)	0.56 (0.21, 1.50)	0.80 (0.30, 2.11)	**2.31 (1.04, 5.12)**	1.13 (0.45, 2.84)
		**Dexmedetomidine**	0.65 (0.27, 1.58)	0.93 (0.39, 2.21)	**2.68 (1.30, 5.54)**	1.31 (0.57, 3.02)
**EA-Emergence Agitation**			**Fentanyl**	1.42 (0.48, 4.22)	**4.10 (1.48, 11.38)**	2.00 (0.70, 5.72)
				**Ketamine**	**2.89 (1.06, 7.86)**	1.41 (0.54, 3.68)
					**Midazolam**	0.49 (0.19, 1.28)
						**Propofol**
**Placebo**	**0.25 (0.09, 0.69)**	**0.43 (0.22, 0.82)**	**3.06 (1.68, 5.58)**	0.96 (0.47, 1.97)	0.40 (0.11, 1.47)	0.64 (0.26, 1.61)
	**Clonidine**	1.68 (0.51, 5.56)	**11.99 (3.73, 38.57)**	**3.76 (1.10, 12.92)**	1.56 (0.60, 4.05)	2.52 (0.65, 9.80)
		**Dexmedetomidine**	**7.13 (2.93, 17.32)**	2.24 (0.92, 5.44)	0.93 (0.22, 4.00)	1.50 (0.49, 4.62)
**PONV-Postoperative Nausea and Vomiting**			**Fentanyl**	**0.31 (0.12, 0.80)**	**0.13 (0.03, 0.55)**	**0.21 (0.08, 0.52)**
				**Ketamine**	0.41 (0.09, 1.84)	0.67 (0.21, 2.15)
					**Midazolam**	1.62 (0.33, 7.98)
						**Propofol**
**Placebo**	0.05 (0.00, 1.25)	**0.09 (0.04, 0.22)**	**0.22 (0.06, 0.75)**	**0.24 (0.07, 0.86)**	**0.08 (0.01, 0.61)**	—
	**Clonidine**	1.72 (0.07, 44.51)	3.99 (0.14, 115.87)	4.48 (0.15, 131.08)	1.51 (0.04, 62.36)	—
		**Dexmedetomidine**	2.32 (0.78, 6.85)	2.60 (0.98, 6.93)	0.88 (0.10, 7.74)	—
**RA-Requiring an Analgesic**			**Fentanyl**	1.12 (0.26, 4.75)	0.38 (0.04, 4.07)	—
				**Ketamine**	0.34 (0.03, 3.58)	—
					**Midazolam**	—
						**Propofol**
**Placebo**	−1.13 (−4.74, 2.48)	**−5.18 (−7.39, −2.97)**	**−6.00 (−9.46, −2.54)**	**−4.82 (−6.72, −2.91)**	—	—
	Clonidine	**−4.04 (−6.91, −1.18)**	−4.87 (−9.87, 0.14)	−3.69 (−7.47, 0.10)	—	—
		**Dexmedetomidine**	−0.82 (−4.93, 3.29)	0.36 (−2.12, 2.84)	—	—
**PAED-Pediatric Anesthesia Emergence Delirium**			**Fentanyl**	1.18 (−2.77, 5.14)	—	—
				**Ketamine**	—	—
					**Midazolam**	—
						**Propofol**
**Placebo**	0.70 (−2.20, 3.60)	**2.02 (0.41, 3.63)**	3.24 (−0.39, 6.88)	0.61 (−1.45, 2.67)	0.75 (−2.30, 3.80)	2.35 (−0.65, 5.36)
	**Clonidine**	1.32 (−2.00, 4.64)	−0.09 (−3.65, 3.47)	0.05 (−4.16, 4.26)	1.65 (−2.52, 5.83)	0.80 (−3.45, 5.05)
		**Dexmedetomidine**	−1.41 (−4.02, 1.20)	−1.27 (−4.73, 2.18)	0.33 (−2.20, 2.86)	−0.52 (−4.02, 2.98)
**Extubation Time**			**Fentanyl**	0.14 (−3.54, 3.82)	1.74 (−1.90, 5.39)	0.89 (−2.84, 4.61)
				**Ketamine**	1.60 (−2.68, 5.89)	0.75 (−3.61, 5.11)
					**Midazolam**	−0.85 (−5.18, 3.47)
						**Propofol**
**Placebo**	−0.85 (−5.88, 4.18)	1.69 (−0.71, 4.08)	−0.69 (−4.05, 2.66)	−0.42 (−5.87, 5.03)	1.54 (−0.14, 3.22)	−1.74 (−7.00, 3.52)
	**Clonidine**	−0.15 (−3.07, 2.78)	1.05 (−3.93, 6.04)	−3.69 (−7.73, 0.36)	2.31 (−1.99, 6.61)	**8.41 (3.83, 12.99)**
		**Dexmedetomidine**	1.20 (−3.48, 5.88)	−3.54 (−7.21, 0.12)	2.46 (−1.49, 6.40)	**8.56 (4.31, 12.81)**
**Emergency Time**			**Fentanyl**	−4.74 (−10.19, 0.71)	1.26 (−4.39, 6.91)	**7.36 (1.50, 13.22)**
				**Ketamine**	**6.00 (1.17, 10.83)**	**12.10 (7.02, 17.18)**
					**Midazolam**	**6.10 (0.81, 11.39)**
						**Propofol**
**Placebo**	**11.00 (3.48, 18.52)**	4.29 (−0.19, 8.76)	**7.31 (2.68, 11.93)**	1.91 (−3.41, 7.22)	10.00 (−0.74, 20.74)	2.03 (−1.90, 5.95)
	**Clonidine**	−6.71 (−15.46, 2.04)	−3.69 (−11.66, 4.28)	−9.09 (−18.28, 0.10)	−1.00 (−14.11, 12.11)	**−8.97 (−17.37, −0.58)**
		**Dexmedetomidine**	3.02 (−3.41, 9.45)	−2.38 (−8.74, 3.98)	5.71 (−5.92, 17.35)	−2.26 (−8.15, 3.62)
**Duration of PACU Stay**			**Fentanyl**	−5.40 (−12.38, 1.58)	2.69 (−9.00, 14.38)	−5.28 (−10.96, 0.39)
				**Ketamine**	8.09 (−3.89, 20.07)	0.12 (−6.03, 6.27)
					**Midazolam**	−7.97 (−19.41, 3.46)
						**Propofol**

*PACU: postanesthesia care unit.

**Table 4:** Relative ranking of six drugs assessed by estimated and predictive probabilities using SUCRA values.

Treatments	Estimated Probabilities							Predictive Probabilities						
	EA	PONV	RA	PAED	Extubation Time	Emergency Time	Duration of PACU Stay	EA	PONV	RA	PAED	Extubation Time	Emergency Time	Duration of PACU Stay
**Placebo**	2.6	28.8	1.3	6.7	**80.7**	**81.2**	**92.2**	7.5	28.8	2.8	8.1	**75.2**	**77.1**	**80.9**
**Clonidine**	56.6	**91.6**	**75.0**	19.8	**58.7**	50.7	12.0	57.2	**91.9**	**74.9**	20.6	**56.9**	51.3	18.2
**Dexmedetomidine**	**66.7**	**73.0**	**74.7**	**73.8**	26.3	**52.8**	51.2	**64.5**	**72.9**	**72.2**	**72.8**	30.7	**53.1**	53.2
**Fentanyl**	**88.8**	0.2	40.7	**83.9**	**61.4**	36.9	29.6	**82.1**	0.2	42.1	**81.6**	**60.0**	38.0	35.2
**Ketamine**	**70.5**	32.9	36.8	**65.7**	57.0	**96.0**	**72.2**	**67.7**	32.6	37.9	**66.8**	56.4	**93.2**	**68.6**
**Midazolam**	16.1	**70.2**	71.3	—	23.8	20.0	21.4	20.0	70.6	**70.2**	—	27.7	21.7	25.9
**Propofol**	49.1	53.3	—	—	42.2	12.4	**71.3**	51.0	53.1	—	—	43.1	15.7	**68.0**

*EA: emergence agitation; PONV: postoperative nausea and vomiting; RA: requiring an analgesic; PAED: pediatric anesthesia emergence delirium; Figures in bold are ranked as the 3 most favorable treatments with respect to different criteria.

## References

[b1] Klastersky J. . A randomized study comparing cisplatin or carboplatin with etoposide in patients with advanced non-small-cell lung cancer: European Organization for Research and Treatment of Cancer Protocol 07861. J Clin Oncol 8, 1556–1562 (1990). 2167953 10.1200/JCO.1990.8.9.1556

[b2] Steinmetz J. . Hemodynamic differences between propofol-remifentanil and sevoflurane anesthesia for repair of cleft lip and palate in infants. Paediatr Anaesth 17, 32–37 (2007). 17184429 10.1111/j.1460-9592.2006.01999.x

[b3] Nakayama S., Furukawa H. & Yanai H. Propofol reduces the incidence of emergence agitation in preschool-aged children as well as in school-aged children: a comparison with sevoflurane. J Anesth 21, 19–23 (2007). 17285408 10.1007/s00540-006-0466-x

[b4] Costi D. . Effects of sevoflurane versus other general anaesthesia on emergence agitation in children. Cochrane Database Syst Rev 9, CD007084 (2014). 10.1002/14651858.CD007084.pub2 PMC10898224 25212274

[b5] Abu-Shahwan I. Effect of propofol on emergence behavior in children after sevoflurane general anesthesia. Paediatr Anaesth 18, 55–59 (2008). 18095967 10.1111/j.1460-9592.2007.02376.x

[b6] Abu-Shahwan I. & Chowdary K. Ketamine is effective in decreasing the incidence of emergence agitation in children undergoing dental repair under sevoflurane general anesthesia. Paediatr Anaesth 17, 846–850 (2007). 17683402 10.1111/j.1460-9592.2007.02298.x

[b7] Akin A. . Dexmedetomidine vs midazolam for premedication of pediatric patients undergoing anesthesia. Paediatr Anaesth 22, 871–876 (2012). 22268591 10.1111/j.1460-9592.2012.03802.x

[b8] Almenrader N. . Premedication in children: a comparison of oral midazolam and oral clonidine. Paediatr Anaesth 17, 1143–1149 (2007). 17986032 10.1111/j.1460-9592.2007.02332.x

[b9] Al-Zaben K. R. . Intraoperative administration of dexmedetomidine reduces the analgesic requirements for children undergoing hypospadius surgery. Eur J Anaesthesiol 27, 247–252 (2010). 19952754 10.1097/EJA.0b013e32833522bf

[b10] Aouad M. T. . A single dose of propofol at the end of surgery for the prevention of emergence agitation in children undergoing strabismus surgery during sevoflurane anesthesia. Anesthesiology 107, 733–738 (2007). 18073548 10.1097/01.anes.0000287009.46896.a7

[b11] Bergendahl H. T. . Clonidine vs. midazolam as premedication in children undergoing adeno-tonsillectomy: a prospective, randomized, controlled clinical trial. Acta Anaesthesiol Scand 48, 1292–1300 (2004). 15504191 10.1111/j.1399-6576.2004.00525.x

[b12] Binstock W. . The effect of premedication with OTFC, with or without ondansetron, on postoperative agitation, and nausea and vomiting in pediatric ambulatory patients. Paediatr Anaesth 14, 759–767 (2004). 15330959 10.1111/j.1460-9592.2004.01296.x

[b13] Bock M. . Comparison of caudal and intravenous clonidine in the prevention of agitation after sevoflurane in children. Br J Anaesth 88, 790–796 (2002). 12173195 10.1093/bja/88.6.790

[b14] Bortone L. . The effect of fentanyl and clonidine on early postoperative negative behavior in children: a double-blind placebo controlled trial. Paediatr Anaesth 24, 614–619 (2014). 24666767 10.1111/pan.12388

[b15] Breschan C. . Midazolam does not reduce emergence delirium after sevoflurane anesthesia in children. Paediatr Anaesth 17, 347–352 (2007). 17359403 10.1111/j.1460-9592.2006.02101.x

[b16] Chen J. Y. . Comparison of the effects of dexmedetomidine, ketamine, and placebo on emergence agitation after strabismus surgery in children. Can J Anaesth 60, 385–392 (2013). 23344921 10.1007/s12630-013-9886-x

[b17] Costi D. . Transition to propofol after sevoflurane anesthesia to prevent emergence agitation: a randomized controlled trial. Paediatr Anaesth 25, 517–523 (2015). 25586124 10.1111/pan.12617

[b18] Cravero J. P., Beach M., Thyr B. & Whalen K. The effect of small dose fentanyl on the emergence characteristics of pediatric patients after sevoflurane anesthesia without surgery. Anesth Analg 97, 364–367, table of contents (2003). 12873918 10.1213/01.ANE.0000070227.78670.43

[b19] Dalens B. J. . Prevention of emergence agitation after sevoflurane anesthesia for pediatric cerebral magnetic resonance imaging by small doses of ketamine or nalbuphine administered just before discontinuing anesthesia. Anesth Analg 102, 1056–1061 (2006). 16551898 10.1213/01.ane.0000200282.38041.1f

[b20] Demirbilek S. . Effects of fentanyl on the incidence of emergence agitation in children receiving desflurane or sevoflurane anaesthesia. Eur J Anaesthesiol 21, 538–542 (2004). 15318465 10.1017/s0265021504007069

[b21] Finkel J. C. . The effect of intranasal fentanyl on the emergence characteristics after sevoflurane anesthesia in children undergoing surgery for bilateral myringotomy tube placement. Anesth Analg 92, 1164–1168 (2001). 11323340 10.1097/00000539-200105000-00016

[b22] Galinkin J. L. . Use of intranasal fentanyl in children undergoing myringotomy and tube placement during halothane and sevoflurane anesthesia. Anesthesiology 93, 1378–1383 (2000). 11149429 10.1097/00000542-200012000-00006

[b23] Ghosh S. M., Agarwala R. B., Pandey M. & Vajifdar H. Efficacy of low-dose caudal clonidine in reduction of sevoflurane-induced agitation in children undergoing urogenital and lower limb surgery: a prospective randomised double-blind study. Eur J Anaesthesiol 28, 329–333 (2011). 21150631 10.1097/EJA.0b013e3283416754

[b24] Guler G. . Single-dose dexmedetomidine reduces agitation and provides smooth extubation after pediatric adenotonsillectomy. Paediatr Anaesth 15, 762–766 (2005). 16101707 10.1111/j.1460-9592.2004.01541.x

[b25] Ibacache M. E., Munoz H. R., Brandes V. & Morales A. L. Single-dose dexmedetomidine reduces agitation after sevoflurane anesthesia in children. Anesth Analg 98, 60–63, table of contents (2004). 14693585 10.1213/01.ANE.0000094947.20838.8E

[b26] Inomata S. . Effects of fentanyl infusion on tracheal intubation and emergence agitation in preschool children anaesthetized with sevoflurane. Br J Anaesth 105, 361–367 (2010). 20627877 10.1093/bja/aeq168

[b27] Isik B., Arslan M., Tunga A. D. & Kurtipek O. Dexmedetomidine decreases emergence agitation in pediatric patients after sevoflurane anesthesia without surgery. Paediatr Anaesth 16, 748–753 (2006). 16879517 10.1111/j.1460-9592.2006.01845.x

[b28] Kain Z. N. . Family-centered preparation for surgery improves perioperative outcomes in children: a randomized controlled trial. Anesthesiology 106, 65–74 (2007). 17197846 10.1097/00000542-200701000-00013

[b29] Kim M. S., Moon B. E., Kim H. & Lee J. R. Comparison of propofol and fentanyl administered at the end of anaesthesia for prevention of emergence agitation after sevoflurane anaesthesia in children. Br J Anaesth 110, 274–280 (2013). 23103775 10.1093/bja/aes382

[b30] Kim N. Y., Kim S. Y., Yoon H. J. & Kil H. K. Effect of dexmedetomidine on sevoflurane requirements and emergence agitation in children undergoing ambulatory surgery. Yonsei Med J 55, 209–215 (2014). 24339309 10.3349/ymj.2014.55.1.209 PMC3874907

[b31] Kulka P. J., Bressem M. & Tryba M. Clonidine prevents sevoflurane-induced agitation in children. Anesth Analg 93, 335–338, 332nd contents page (2001). 11473855

[b32] Lankinen U., Avela R. & Tarkkila P. The prevention of emergence agitation with tropisetron or clonidine after sevoflurane anesthesia in small children undergoing adenoidectomy. Anesth Analg 102, 1383–1386 (2006). 16632814 10.1213/01.ane.0000205745.84044.31

[b33] Lee C. J. . The effect of propofol on emergence agitation in children receiving sevoflurane for adenotonsillectomy. Korean J Anesthesiol 59, 75–81 (2010). 20740210 10.4097/kjae.2010.59.2.75 PMC2926433

[b34] Lee Y. S. . The effect of ketamine on the incidence of emergence agitation in children undergoing tonsillectomy and adenoidectomy under sevoflurane general anesthesia. Korean J Anesthesiol 58, 440–445 (2010). 20532051 10.4097/kjae.2010.58.5.440 PMC2881518

[b35] Lili X., Jianjun S. & Haiyan Z. The application of dexmedetomidine in children undergoing vitreoretinal surgery. J Anesth 26, 556–561 (2012). 22415678 10.1007/s00540-012-1354-1

[b36] Lundblad M., Marhofer D., Eksborg S. & Lonnqvist P. A. Dexmedetomidine as adjunct to ilioinguinal/iliohypogastric nerve blocks for pediatric inguinal hernia repair: an exploratory randomized controlled trial. Paediatr Anaesth 25, 897–905 (2015). 26095747 10.1111/pan.12704

[b37] Meng Q. T. . Dexmedetomidine reduces emergence agitation after tonsillectomy in children by sevoflurane anesthesia: a case-control study. Int J Pediatr Otorhinolaryngol 76, 1036–1041 (2012). 22537843 10.1016/j.ijporl.2012.03.028

[b38] Ozcengiz D., Gunes Y. & Ozmete O. Oral melatonin, dexmedetomidine, and midazolam for prevention of postoperative agitation in children. J Anesth 25, 184–188 (2011). 21327805 10.1007/s00540-011-1099-2

[b39] Patel A. . Dexmedetomidine infusion for analgesia and prevention of emergence agitation in children with obstructive sleep apnea syndrome undergoing tonsillectomy and adenoidectomy. Anesth Analg 111, 1004–1010 (2010). 20705788 10.1213/ANE.0b013e3181ee82fa

[b40] Pestieau S. R. . The effect of dexmedetomidine during myringotomy and pressure-equalizing tube placement in children. Paediatr Anaesth 21, 1128–1135 (2011). 21575102 10.1111/j.1460-9592.2011.03615.x

[b41] Rampersad S. . Two-agent analgesia versus acetaminophen in children having bilateral myringotomies and tubes surgery. Paediatr Anaesth 20, 1028–1035 (2010). 20964769 10.1111/j.1460-9592.2010.03427.x PMC4005868

[b42] Saadawy I. . Effect of dexmedetomidine on the characteristics of bupivacaine in a caudal block in pediatrics. Acta Anaesthesiol Scand 53, 251–256 (2009). 19076110 10.1111/j.1399-6576.2008.01818.x

[b43] Sato M. . Effect of single-dose dexmedetomidine on emergence agitation and recovery profiles after sevoflurane anesthesia in pediatric ambulatory surgery. J Anesth 24, 675–682 (2010). 20585813 10.1007/s00540-010-0976-4

[b44] Sheta S. A., Al-Sarheed M. A. & Abdelhalim A. A. Intranasal dexmedetomidine vs midazolam for premedication in children undergoing complete dental rehabilitation: a double-blinded randomized controlled trial. Paediatr Anaesth 24, 181–189 (2014). 24237879 10.1111/pan.12287

[b45] Shukry M., Clyde M. C., Kalarickal P. L. & Ramadhyani U. Does dexmedetomidine prevent emergence delirium in children after sevoflurane-based general anesthesia? Paediatr Anaesth 15, 1098–1104 (2005). 16324031 10.1111/j.1460-9592.2005.01660.x

[b46] Tazeroualti N. . Oral clonidine vs midazolam in the prevention of sevoflurane-induced agitation in children. a prospective, randomized, controlled trial. Br J Anaesth 98, 667–671 (2007). 17416907 10.1093/bja/aem071

[b47] Tesoro S., Mezzetti D., Marchesini L. & Peduto V. A. Clonidine treatment for agitation in children after sevoflurane anesthesia. Anesth Analg 101, 1619–1622 (2005). 16301230 10.1213/01.ANE.0000184204.81877.53

[b48] Tsai P. S. . Ketamine but not propofol provides additional effects on attenuating sevoflurane-induced emergence agitation in midazolam premedicated pediatric patients. Paediatr Anaesth 18, 1114–1115 (2008). 18950343 10.1111/j.1460-9592.2008.02593.x

[b49] Viitanen H., Annila P., Viitanen M. & Tarkkila P. Premedication with midazolam delays recovery after ambulatory sevoflurane anesthesia in children. Anesth Analg 89, 75–79 (1999). 10389782 10.1097/00000539-199907000-00014

[b50] Fang X. Z. . Network Meta-Analysis on the Efficacy of Dexmedetomidine, Midazolam, Ketamine, Propofol, and Fentanyl for the Prevention of Sevoflurane-Related Emergence Agitation in Children. Am J Ther (2015). 10.1097/MJT.0000000000000321 26186683

[b51] Su F. & Hammer G. B. Dexmedetomidine: pediatric pharmacology, clinical uses and safety. Expert Opin Drug Saf 10, 55–66 (2011). 20718689 10.1517/14740338.2010.512609

[b52] Farag E. . The use of dexmedetomidine in anesthesia and intensive care: a review. Curr Pharm Des 18, 6257–6265 (2012). 22762468 10.2174/138161212803832272

[b53] Kim D. . Effect of ketorolac on the prevention of emergence agitation in children after sevoflurane anesthesia. Korean J Anesthesiol 64, 240–245 (2013). 23560190 10.4097/kjae.2013.64.3.240 PMC3611074

[b54] Cravero J., Surgenor S. & Whalen K. Emergence agitation in paediatric patients after sevoflurane anaesthesia and no surgery: a comparison with halothane. Paediatr Anaesth 10, 419–424 (2000). 10886700 10.1046/j.1460-9592.2000.00560.x

[b55] Dahmani S. . Pharmacological prevention of sevoflurane- and desflurane-related emergence agitation in children: a meta-analysis of published studies. Br J Anaesth 104, 216–223 (2010). 20047899 10.1093/bja/aep376

[b56] Sun L., Guo R. & Sun L. Dexmedetomidine for preventing sevoflurane-related emergence agitation in children: a meta-analysis of randomized controlled trials. Acta Anaesthesiol Scand 58, 642–650 (2014). 24588393 10.1111/aas.12292

[b57] Sanders R. D. . Dexmedetomidine attenuates isoflurane-induced neurocognitive impairment in neonatal rats. Anesthesiology 110, 1077–1085 (2009). 19352168 10.1097/ALN.0b013e31819daedd

[b58] Engelhard K. . The effect of the alpha 2-agonist dexmedetomidine and the N-methyl-D-aspartate antagonist S(+)-ketamine on the expression of apoptosis-regulating proteins after incomplete cerebral ischemia and reperfusion in rats. Anesth Analg 96, 524–531, table of contents (2003). 12538207 10.1097/00000539-200302000-00041

[b59] Wang Q., Lu R., Zhao J. & Limbird L. E. Arrestin serves as a molecular switch, linking endogenous alpha2-adrenergic receptor to SRC-dependent, but not SRC-independent, ERK activation. J Biol Chem 281, 25948–25955 (2006). 16809338 10.1074/jbc.M605415200

[b60] Cohen I. T. . The effect of fentanyl on the emergence characteristics after desflurane or sevoflurane anesthesia in children. Anesth Analg 94, 1178–1181, table of contents (2002). 11973185 10.1097/00000539-200205000-00023

[b61] Veyckemans F. Excitation phenomena during sevoflurane anaesthesia in children. Curr Opin Anaesthesiol 14, 339–343 (2001). 17019113 10.1097/00001503-200106000-00010

[b62] Shi F. . Effects of Fentanyl on Emergence Agitation in Children under Sevoflurane Anesthesia: Meta-Analysis of Randomized Controlled Trials. PLoS One 10, e0135244 (2015). 26275039 10.1371/journal.pone.0135244 PMC4537096

[b63] Warncke T., Stubhaug A. & Jorum E. Ketamine, an NMDA receptor antagonist, suppresses spatial and temporal properties of burn-induced secondary hyperalgesia in man: a double-blind, cross-over comparison with morphine and placebo. Pain 72, 99–106 (1997). 9272793 10.1016/s0304-3959(97)00006-7

[b64] Honarmand A., Safavi M. R. & Jamshidi M. The preventative analgesic effect of preincisional peritonsillar infiltration of two low doses of ketamine for postoperative pain relief in children following adenotonsillectomy. A randomized, double-blind, placebo-controlled study. Paediatr Anaesth 18, 508–514 (2008). 18312522 10.1111/j.1460-9592.2008.02461.x

[b65] Dahmani S. . Ketamine for perioperative pain management in children: a meta-analysis of published studies. Paediatr Anaesth 21, 636–652 (2011). 21447047 10.1111/j.1460-9592.2011.03566.x

[b66] White P. F., Way W. L. & Trevor A. J. Ketamine–its pharmacology and therapeutic uses. Anesthesiology 56, 119–136 (1982). 6892475 10.1097/00000542-198202000-00007

