# Supplementary material for: Efficacy and Acceptability of Different Auxiliary Drugs in Pediatric Sevoflurane Anesthesia: A Network Meta-analysis of Mixed Treatment Comparisons
Source: Sci Rep. 2016 Nov 10;6:36553. doi: 10.1038/srep36553 (PMC5103214; doi:10.1038/srep36553)
Supplement: Supplementary Information [file srep36553-s1.pdf]

Efficacy and Acceptability of Different Auxiliary Drugs in Pediatric Sevoflurane Anesthesia: A Network Meta-analysis of Mixed Treatment Comparisons

Running title: Auxiliary drugs in pediatric sevoflurane anesthesia

Wuchao Wang*, Panchuan Huang, Weiwei Gao, Fangli Cao, Mingling Yi, Liyong Chen, Xiaoli Guo*

Supplementary Figure Legends

Figure S1 (A) The forest plot of different treatment on postoperative nausea and vomiting from network meta-analysis; (B) The cumulative ranking probabilities of different treatment on postoperative nausea and vomiting

Figure S2 (A) The forest plot of different treatment on number of patients requiring an analgesic from network meta-analysis; (B) The cumulative ranking probabilities of different treatment on patients requiring an analgesic

Figure S3 (A) The forest plot of different treatment on pediatric anesthesia emergence delirium from network meta-analysis; (B) The cumulative ranking probabilities of different treatment on pediatric anesthesia emergence delirium

Figure S4 (A) The forest plot of different treatment on extubation time from network meta-analysis; (B) The cumulative ranking probabilities of different treatment on extubation time

Figure S5 (A) The forest plot of different treatment on emergency time from network meta-analysis; (B) The cumulative ranking probabilities of different treatment on emergency time

Figure S6 (A) The forest plot of different treatment on duration of postanesthesia care unit stay from network meta-analysis; (B) The cumulative ranking probabilities of different treatment on duration of postanesthesia care unit stay

Table S1 QUORUM statement checklist

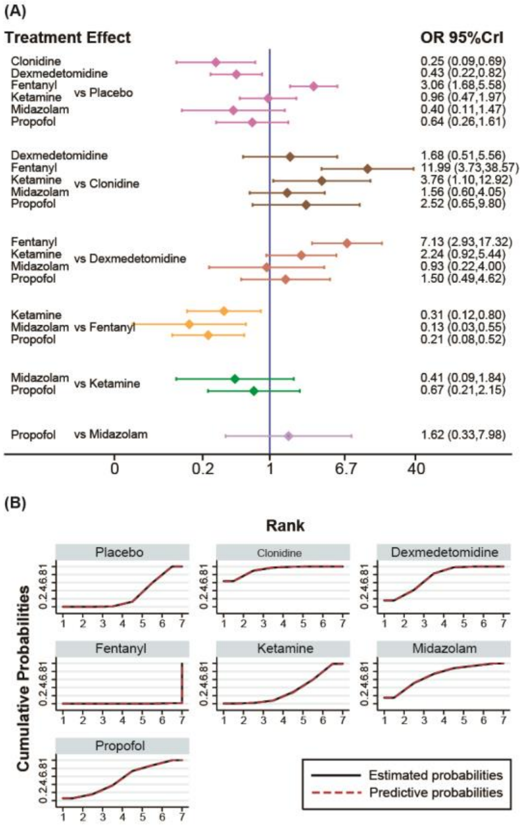

Figure S1 (A) The forest plot of different treatment on postoperative nausea and vomiting from network meta-analysis; (B) The cumulative ranking probabilities of different treatment on postoperative nausea and vomiting

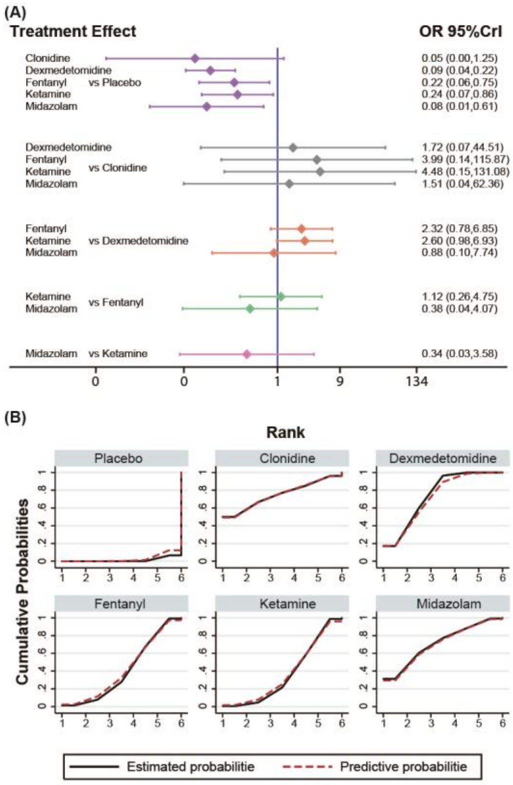

Figure S2 (A) The forest plot of different treatment on number of patients requiring an analgesic from network meta-analysis; (B) The cumulative ranking probabilities of different treatment on patients requiring an analgesic

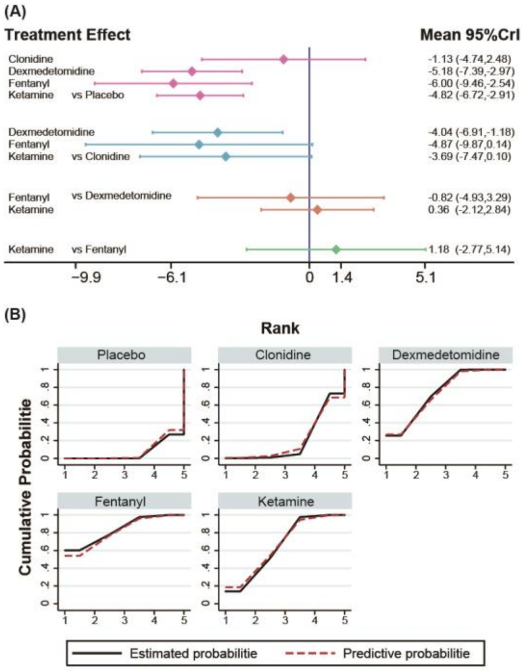

Figure S3 (A) The forest plot of different treatment on pediatric anesthesia emergence delirium from network meta-analysis; (B) The cumulative ranking probabilities of different treatment on pediatric anesthesia emergence delirium

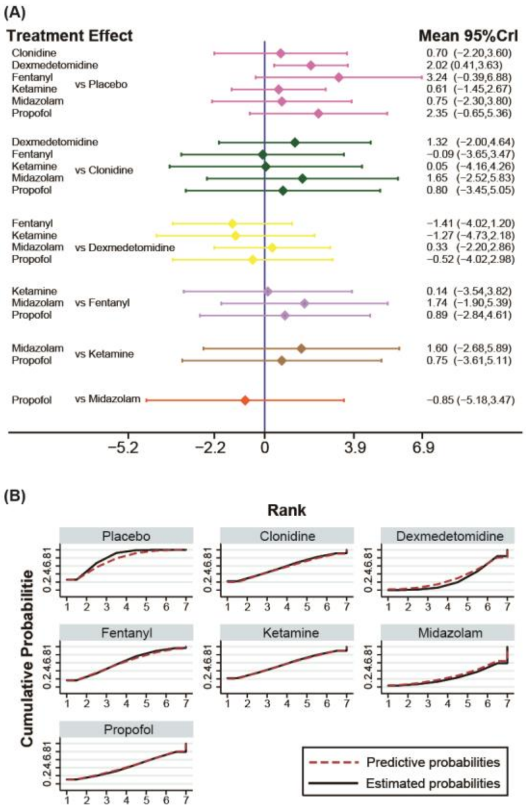

Figure S4 (A) The forest plot of different treatment on extubation time from network meta-analysis; (B) The cumulative ranking probabilities of different treatment on extubation time

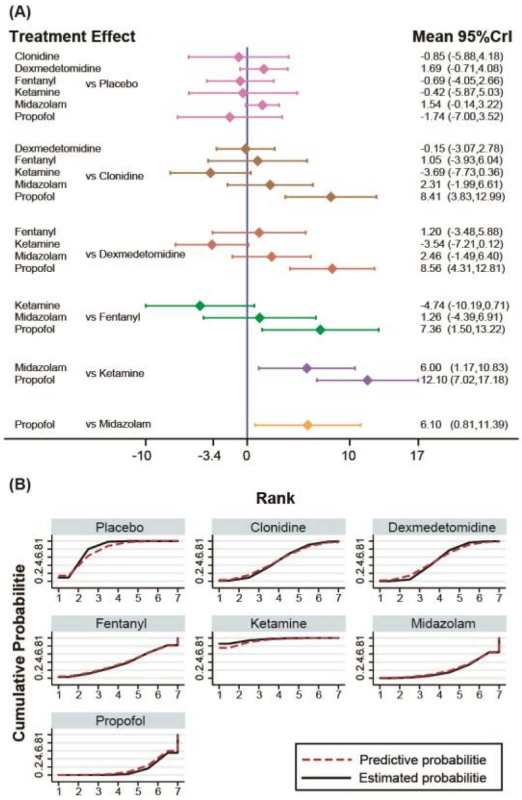

Figure S5 (A) The forest plot of different treatment on emergency time from network meta-analysis; (B) The cumulative ranking probabilities of different treatment on emergency time

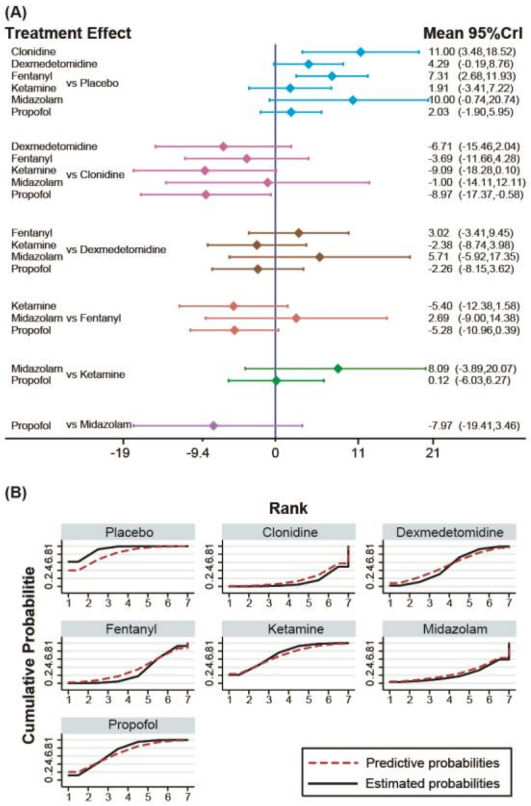

Figure S6 (A) The forest plot of different treatment on duration of postanesthesia care unit stay from network meta-analysis; (B) The cumulative ranking probabilities of different treatment on duration of postanesthesia care unit stay

Table S1. A Jadad Scale concerning randomization, blinding and withdraw of the included papers

ID, author, year	Randomized	Blinded	Withdrawal
01 Lundblad 2015	2	2	1
02 Costi 2015	2	2	1
03 Sheta 2014	2	2	1
04 Kim 2014	2	2	1
05 Bortone 2014	2	2	0
06 Kim 2013	2	2	1
07 Chen 2013	2	2	1
08 Meng 2012	2	2	1
09 Lili 2012	2	2	0
10 Akin 2012	2	2	0
11 Pestieau 2011	2	2	1
12 Ozcengiz 2011	2	2	0
13 Ghosh 2011	2	2	0
14 Sato 2010	2	2	0
15 Rampersad 2010	2	2	1
16 Patel 2010	2	2	0
17 Lee 2010	2	2	0
18 Lee 2010	2	2	0
19 Inomata 2010	2	2	1
20 Al-Zaben 2010	2	2	0
21 Saadawy 2009	2	2	1
22 Tsai 2008	2	2	0
23 Abu-Shahwan 2008	2	2	0
24 Tazeroualti 2007	2	2	0
25 Kain 2007	2	1	1
26 Breschan 2007	2	1	0
27 Aouad 2007	2	2	0
28 Almenrader 2007	2	0	0
29 Alu-Shahwan 2007	2	2	0
30 Lankinen 2006	1	2	0
31 Isik 2006	1	2	0
32 Dalens 2006	1	2	0
33 Tesoro 2005	1	2	0
34 Shukry 2005	1	2	0
35 Guler 2005	2	2	0
36 Ibacache 2004	1	2	0
37 Demirbilek 2004	0	0	0
38 Binstock 2004	2	2	0
39 Bergendahl 2004	2	2	0
40 Cravero 2003	2	2	0
41 Bock 2002	1	1	0
42 Kulka 2001	2	2	0
43 Finkel 2001	2	2	0
44 Galinkin 2000	2	2	0
45 Viitanen 1999	2	2	0
